# Parasitoidism of Dermaptera by Braconidae (Hymenoptera) confirmed

**DOI:** 10.3897/zookeys.1268.181472

**Published:** 2026-02-04

**Authors:** Yuan-Cheng Hsu, Dávid Rédei

**Affiliations:** 1 Department of Entomology, National Chung Hsing University, Taichung 40227, Taiwan Department of Entomology, National Chung Hsing University Taichung Taiwan

**Keywords:** Bionomics, earwigs, non-native species, Oriental Region, Palaearctic Region, parasitoid

## Abstract

*Orionis coxator* (Belokobylskij, 1995) (Hymenoptera: Braconidae: Euphorinae: Perilitini) was reared from an individual of *Apterygida tuberculosa* Shiraki, 1928 (Dermaptera: Forficulidae) in Taiwan. This represents a new record of *O. coxator* and the genus *Orionis* Shaw, 1987 from Taiwan, as well as the first confirmed case of a hymenopteran species as a primary parasitoid of a dermapteran. The male of *O. coxator* is redescribed and illustrated, and its diagnostic characters are discussed. Published records of parasitoidism of Ichneumonoidea and other Hymenoptera in Dermaptera and other Polyneoptera are briefly reviewed, and some biological aspects of parasitoidism of braconids in *A. tuberculosa* and other species of Dermaptera in Taiwan are discussed.

## Introduction

Parasitoidism is the most prevalent strategy in Hymenoptera. It is estimated that about 70% of the described hymenopteran species are parasitoids; it is the ancestral state for the clade Apocrita+Orussoidea (Blaimer et al. 2023). Although parasitoid hymenopterans consume a broad range of insect groups, and rarely other arthropods (e.g., spiders, mites) as well, with highly diverse feeding strategies (Gauld and Bolton 1988; Godfray 1994; Quicke 1997; Whitfield 2003), no hymenopteran species have been recorded to parasitize Dermaptera until a most recent report (Bendixen and Shaw 2024) that provided a photo of a female of *Orionis coxator* (Belokobylskij, 1995) (Braconidae: Euphorinae) apparently ovipositing into an adult female *Apterygida albipennis* (Mühlfeld, 1825) (Dermaptera: Forficulidae) in northern Germany, and suspected that members of this order may indeed serve as hosts of at least this braconid species, and potentially also other species of *Orionis* Shaw, 1987.

In the present paper we confirm parasitoidism of *Orionis coxator* in another species of the same earwig genus, *Apterygida tuberculosa* Shiraki, 1928 (endemic to Taiwan), based on rearing of field-collected host individuals in the laboratory. The genus *Orionis* and *O. coxator* are recorded as new to Taiwan, the male of this species is redescribed and illustrated, and the diagnostic characters for *Orionis* and *O. coxator* are discussed.

## Material and methods

Earwig individuals were kept separated in Petri dishes and other small containers; parasitoids emerging from them were allowed to pupate in the same enclosure. Emerged adult parasitoids were killed about two days after their emergence.

**Figures 1–5.**
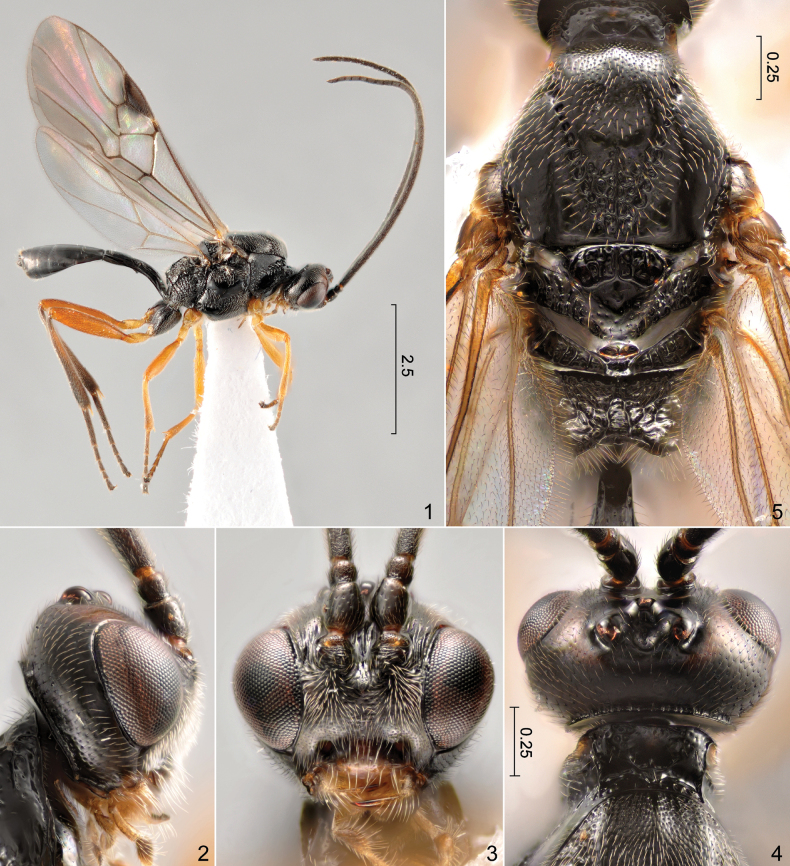
*Orionis coxator* (Belokobylskij, 1995), male. **1**. Habitus; **2**. Head, lateral view; **3**. Same, frontal view; **4**. Same, dorsal view; **5**. Mesosoma, dorsal view. Scale bars in mm.

Specimens were examined using a stereoscopic microscope (Leica M205C). Measurements were taken using a calibrated ocular micrometer. The habitus photograph (Fig. 1) was taken with a Nikon D90 camera equipped with an AF-S Micro Nikkor 60mm f/2.8G ED lens, the rest of the images with the same camera equipped with a Nikon E Plan 4×/0.10 or a Nikon Plan 10×/0.25 lens set on a Nikkor 200mm f/4 AI teleobjective; images were stacked using the software Helicon Focus 8.2.2. Morphological terminology follows van Achterberg (1993).

## Results

### Orionis coxator

Taxon classificationAnimaliaHymenopteraBraconidae

(Belokobylskij, 1995)

FA710FFD-A5F9-5E70-89EF-86AAC6787B08

Figs 1, 2, 3, 4, 5, 6, 7, 8, 9, 10, 11

Perilitus coxator Belokobylskij, 1995: 302. Holotype: ♀, Russia: Kunashir Is., Yushno-Kurilsk; deposited in Zoological Institute, Russian Academy of Sciences, St. Petersburg.
Perilitus (Perilitus) coxator : Belokobylskij (2000a: 100) (diagnostic characters), Belokobylskij (2000b: 285) (in key, figures, distribution), Ku et al. (2001: 142) (records, distribution).
Perilitus coxator : Stigenberg et al. (2015: 576, 578) (phylogenetic analysis), Anonymous (2019: 750) (listed).
Orionis coxator : Broad and Stigenberg (2021: 136, 137, 139) (in key, generic placement, diagnosis, photos, record, distribution, ecology), Fujie (2022: 403, 405, 410) (photos), Fujie and Maeto (2022: 258) (diagnostic characters, records, distribution), Gupta et al. (2024: 225, 236) (diagnostic characters, in key), Libert and van Achterberg (2024: 110) (records).
Perilitus erratus (non Chen & van Achterberg, 1997): van Achterberg et al. (2020: 163) (diagnosis, photos, record, distribution). Probable misidentification (Broad and Stigenberg 2021: 137).

#### Material examined.

Host individual (*Apterygida tuberculosa* Shiraki, 1928) collected: Taiwan • 1 ♂, Taichung, Hoping Distr., Fushoushan Tea Plantation, 24.235°N, 121.244°E, 2320 m a.s.l., 13 Jan. 2025, Y.-C. Wu leg. Cocoon appeared 23 Jan. 2025; adult *Orionis coxator* (♂) (Fig. 1) emerged 13 Feb. 2025. Host and braconid individuals are both preserved in the collection of the Department of Entomology, National Chung Hsing University, Taichung, Taiwan.

**Figures 6–12.**
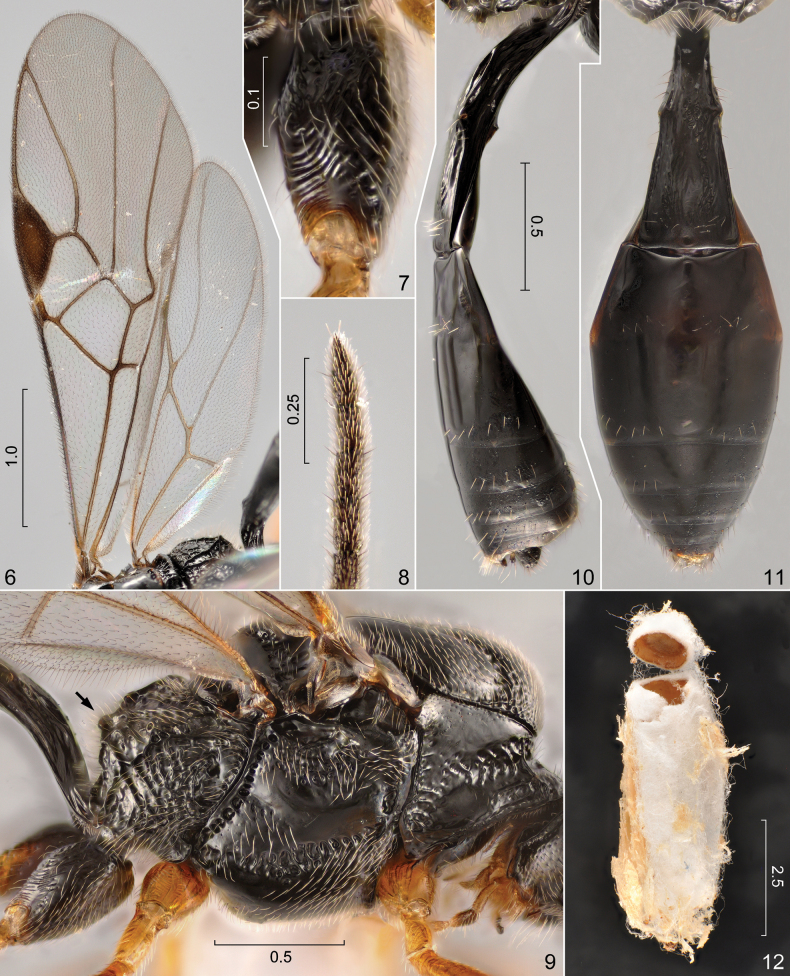
*Orionis coxator* (Belokobylskij, 1995), male. **6**. Right wings, dorsal view; **7**. Right hind coxa, anterodorsal view; **8**. Distal portion of right antenna; **9**. Mesosoma, lateral view; **10**. Metasoma, lateral view; **11**. Same, dorsal view; **12**. Cocoon. Scale bars in mm.

#### Description of male.

***Colour***. Body black; vertex extensively brown laterad of lateral ocelli; clypeus (except dorsal portion), mandible (except darkened tip), and maxillary palp (except brownish second palpomere) stramineous; antenna brownish black; tegulae blackish brown, laterally yellowish; wings smoky brown, pterostigma brown, veins of fore wing dark brown proximally, pale brown distally, veins of hind wing pale brown; legs pale reddish yellow, fore and mid tarsi gradually darkened towards apex, hind coxa black, hind femur orange, hind tibia and tarsus dark brown.

***Head*** (Figs 2–4): in dorsal view eye 1.15 times as long as temple; OOL (distance between lateral ocellus and ipsilateral eye): OD (largest diameter of one of the posterior ocelli): POL (distance between posterior ocelli) = 22: 10: 35; face distinctly wider than long, about 0.45 times as broad as maximum width of head across eyes; clypeus about 2.2 times as wide as long, almost horizontally truncate ventrally; length of malar space subequal to basal width of mandible. ***Antenna***: flagellum with 28 flagellomeres, first flagellomere 1.2 times as long as second flagellomere, first to third flagellomeres 2.0, 1.7 and 1.6 times longer than wide, respectively. ***Mesosoma*** (Figs 5, 9) 1.65 times as long as high; anterior portion of propodeum more strongly declivous and dorsally more flattened than in female, dorsal outline in lateral view abruptly becoming nearly vertical around middle of its length, with a pair of submedian tubercle-like projections between declivous anterior and subvertical posterior portion (Fig. 9: arrow). ***Wings*** (Fig. 6) as in female. ***Legs***. Hind coxa coarsely punctured proximally, deeply transversely rugose distally, rugae strongly curved on dorsal surface (Figs 7, 9); hind femur, tibia and basitarsus 4.4, 8.7 and 6.7 times longer than wide, respectively. ***Metasoma*** (Figs 10, 11) subequal in length to head and prosoma combined; syntergum II+III about 1.1 times as broad as long, with faint but traceable intersegmental boundary around its middle; tergum III gradually broadening with lateral margins, lateral outline obliquely angulate between terga II and III, tergum III narrowed starting from its anterior margin.

***Measurements*** (in mm; 1 male). Body length 4.60; length of head (in dorsal view) 0.65, width 1.03; length of mesosoma 1.81, width of mesonotum 0.94; length of fore wing 4.13; length of hind wing 3.06; length of metasoma 2.28, width 0.77.

#### Description of cocoon.

White, oval, formed by densely packed silk fibres, covered by a thin layer of loose, disordered fibres that incorporate substrate (wood dust) particles of the environment, attached to a piece of wood in the enclosure of the host (removed for photographing) (Fig. 12). Length 6.48 mm, greatest width 2.52 mm.

#### Distribution.

The species was described from the Far East territories of Russia and subsequently reported from surrounding regions; East Asia might therefore be its native area of distribution. It has recently been reported from several localities in western Europe, where it is probably introduced (Broad and Stigenberg 2021). Published records of the species can be summarized as follows.

RUSSIA. Primorsky Krai, Sakhalin Obl., Kuril Islands (Kunashir Is., Shikotan Is.) (Belokobylskij 1995, 2000b). — SOUTH KOREA. Jeollanam-do; Gyeongsangbuk-do; Gangwon-do; Daegu; Jeju Is. (Ku et al. 2001). — JAPAN. Hokkaidô; Honshû; Shikoku (Fujie and Maeto 2022). — TAIWAN. Taichung (new record). — MYANMAR (Belokobylskij 2000b). — UNITED KINGDOM. Kent; Hampshire? (Broad and Stigenberg 2021) (introduced?). — THE NETHERLANDS. Amsterdam (van Achterberg et al. 2020, as *Perilitus erratus*) (introduced?). — BELGIUM. Somal (Libert and van Achterberg 2024) (introduced?). — GERMANY. Schleswig-Holstein: Mohrkirch (Bendixen and Shaw 2024) (introduced?).

## Discussion

### Identification and intraspecific variability in *Orionis coxator*

Based on the original description of *Orionis coxator* and accompanying figures (Belokobylskij 1995), the key of Belokobylskij (2000b) and Gupta et al. (2024), and photographs of females published by van Achterberg et al. (2020) (as *Perilitus erratus*, in error?, cf. Broad and Stigenberg 2021) and Broad and Stigenberg (2021), the male examined in course of this study differs from females of *O. coxator* at least in the following aspects: (1) face distinctly broader, mesal outline of eyes weakly converging ventrally (Fig. 3); (2) anterior portion of dorsal surface of propodeum more strongly declivous and more flattened, submedially with a pair of tubercle-like projections between the declivous anterior and the subvertical posterior portion, at the two sides of the median longitudinal depression (Fig. 9: arrow); and (3) hind tibiae strongly suffused with dark brown in most of its length, contrasting with orange hind femur (Fig. 1). The first was already noted by Belokobylskij (1995: 304); see further in the next paragraph. The third is probably a mere intraspecific variation; similar differences in the extent of pigmentation of different body parts are common in braconids. Although Belokobylskij (1995) claimed that the hind coxae of all males of *O. coxator* examined by him were “pale reddish brown, only basally dark”; the hind coxae of our male are black, like females of *O. coxator*; this character is probably also subject to considerable intraspecific variability. Despite the above mentioned differences, the male before us closely agrees with all important morphological characters of females of *O. coxator*, particularly the characteristic sculpture of the hind coxae (Figs 7, 9), the postfurcal recurrent vein of the fore wing (Fig. 6), and the relatively short first flagellomere; therefore, we consider them conspecific.

The ventrally strongly convergent mesal margins of the eyes were mentioned by Broad and Stigenberg (2021), Fujie (2022) and Fujie and Maeto (2022) as a diagnostic character for the genus *Orionis*. *Orionis coxator* exhibits a marked sexual dimorphism in this respect, the male having a much broader face and ventrally only weakly converging eyes (Fig. 3). The aforementioned character is probably mainly true only for female individuals of *Orionis* spp.

### The distribution of *O. coxator* and its occurrence in Taiwan

*Orionis coxator* exhibits a broadly disjunct area, with records in East Asia and in western Europe. Broad and Stigenberg (2021) convincingly argued that this unusual pattern of distribution suggests that the European population is the result of a recent introduction. It is nevertheless remarkable that the species has also remained undetected in Japan, a country where braconid wasps have been a subject of relatively active study for about a century, until most recently (Belokobylskij 2000b; Fujie and Maeto 2022).

We are not aware of any records of the species from China, but judging from its distribution in the region, it likely occurs in the country. Reports of *Orionis erratus* (Chen & van Achterberg, 1997) (formerly *Perilitus erratus*) from Liaoning, Shaanxi, Guizhou and Yunnan (Chen and van Achterberg 1997; Chen et al. 2004; van Achterberg et al. 2020) might at least partly pertain to this species. The record from Myanmar (Belokobylskij 2000b, without a more specific locality mentioned) suggests that the species may be more widely distributed in eastern Asia than currently thought.

The occurrence of the species in Taiwan, reported for the first time in this paper (at the same time also representing the first record of the genus *Orionis*), remains puzzling as well. Although the possibility of a recent introduction cannot be excluded, some circumstances apparently indicate that *O. coxator* is native to Taiwan. These include the proximity of Taiwan to reported East Asian localities of *O. coxator* and the occurrence of the species in a montane locality at an altitude of 2320 m, far from significant human settlements, in a dermapteran species that is not only endemic to Taiwan but highly restricted in its geographic range even within the island (only occurring in high-altitude regions).

### Ichneumonoidea species parasitizing Dermaptera

Except for the above mentioned report of Bendixen and Shaw (2024), apparently no species of Ichneumonoidea has been reported to parasitize members of the order Dermaptera. Most members of Ichneumonidae are restricted to endopterygote larvae, and the vast majority of braconids also consume endopterygotan hosts (Quicke 1997, 2015). Euphorinae seems to be the most diverse braconid subfamily in respect of host repertoire, comprising species that parasitize members of adult and nymphal Orthoptera, adult and nymphal Psocoptera, adult and nymphal Hemiptera (Heteroptera), adult Neuroptera, adult (rarely larval) Coleoptera and adult Hymenoptera (Apocrita) (Shaw 1985, 1988; Maetô 1990; Shaw and Huddleston 1991; Quicke 2015; Belokobylskij 2018).

There are only very few reported cases of braconids parasitizing polyneopterans, including the following:

- *Sericobracon arimaensis* Shaw, 1985 (Doryctinae), apparently a solitary idiobiont endoparasitoid of adults of *Antipaluria urichi* (Saussure, 1896) (Embioptera: Clothodidae) (Shaw and Edgerly 1985).
- *Katytermus palmicola* van Achterberg, 1996 (Lysiterminae, but included in Rogadinae, Hormiinae or Exothecinae by some authors), apparently a gregarious koinobiont endoparasitoid reared from larvae of an unidentified species of Orthoptera: Ensifera (probably belonging to Gryllacrididae) in Malaysia: Selangor (van Achterberg and Steiner 1996).
- *Notioperilitus morabinarum* (Blackith, 1967) (Euphorinae: Perilitini), a solitary koinobiont endoparasitoid reared on multiple occasions from adults of different unidentified species of grasshoppers belonging to Eumastacidae: Morabinae (an endemic Australian subfamily) in Australia (Blackith 1967; Shaw 1988; Belokobylskij 2018).
- Possibly also members of Doryctinae: Ypsistocerini, occurring in termite nests, possibly parasitizing termites (Shaw and Huddleston 1991) or rather larvae of inquiline coleopterans (van Achterberg 1995).

Of the aforementioned cases, *Sericobracon arimaensis*, Ypsistocerini and *Katytermus palmicola* are taxonomically unrelated to *Orionis coxator*, and their known bionomics are also highly different. *Notioperilitus morabinarum* is, however, taxonomically closely related to *Orionis* spp. (both genera belonging to the tribe Perilitini). Other members of Perilitini parasitize adults (rarely larvae) of several families of Coleoptera, mainly Chrysomelidae and Cerambycidae (Tobias 1965, 1966; Shaw 1985, 1988; Belokobylskij 2018). *Notioperilitus morabinarum* probably represents a shift from a (larval?) coleopteran to an orthopteran host (Shaw 1988; Belokobylskij 2018); *O. coxator* probably represents another, independent case of host shift from (larval?) Coleoptera to Dermaptera.

### Other hymenopteran groups parasitizing Dermaptera

We are not aware of any published reports of hymenopterans parasitizing any species of Dermaptera, either. Quicke (1997: 366) indicated that Dermaptera might be minor hosts of some species of Pteromalidae. This probably pertains to *Dibrachys microgastri* (Bouché, 1834) [formerly frequently cited as *Dibrachys cavus* (Walker, 1835), the latter name now being a junior synonym (Peters and Baur 2011)], which has indeed been listed as a parasitoid of *Forficula auricularia* Linnaeus, 1758 (Forficulidae) by various catalogues (Peck 1963: 682; Krombein et al. 1979: 826). These entries, however, are apparently based on primary literature reporting this widely polyphagous species hyperparasitizing *F. auricularia* through *Triarthra setipennis* (Fallén, 1810) (Diptera: Tachinidae) (earlier frequently cited as *Digonochaeta setipennis*), a tachinid that is fairly common in northern and western Europe and develops in *F. auricularia* and some other earwig species (Davies 1927; Thompson 1928; Carl and Zwölfer 1965; Herting 1971, 1978; Vidal 1997; Peters and Baur 2011). Accordingly, *Orionis coxator* appears to be the first confirmed hymenopteran species to develop in a dermapteran primary host species.

### Parasitoidism of Braconidae in Dermaptera

Our results support that *Orionis coxator* is indeed a koinobiont primary endoparasitoid of dermapteran host(s), as suggested by Bendixen and Shaw (2024) based on an observation of a female ovipositing in *Apterygida albipennis*.

During the past years, the first author observed parasitoidism of two additional species of Dermaptera by hymenopterans. In the first case, parasitoids were reared from an adult male of *Cranopygia sauteri* (Burr, 1912) (Pygidicranidae) collected in central Taiwan (Nantou County) in 2021. Approximately 20 wasp larvae emerged from the end of the abdomen and spun cocoons within a day; most of them developed to adult braconids 15 days later. In the second case, a single cocoon was found in the rearing container of a female *Timomenus aeris* (Shiraki, 1905) (Forficulidae) collected in western Taiwan (Miaoli County). The earwig exhibited obvious guarding behaviour for nine days, then ceased attending the cocoon, and two days later an adult braconid emerged from the cocoon. The importance of these findings was not realized at that time, and therefore, the parasitoid individuals were not kept and not examined further. These observations suggest that parasitoidism of Braconidae in Dermaptera may be more common than currently thought. In all three observed cases of braconids parasitizing dermapterans reported in this paper, the hosts remained remarkably active and vigorous after the wasp larvae left their body, and survived for more than two weeks. Such a long survival time is highly unusual among ichneumonoids (Quicke 1997, 2015). The interaction between braconids and dermapterans needs further study.

## Supplementary Material

XML Treatment for Orionis coxator

## Additional information

### Use of AI

No use of AI was reported.

### Author ORCIDs

Yuan-Cheng Hsu https://orcid.org/0009-0004-3478-615X

Dávid Rédei https://orcid.org/0000-0003-1550-2110

### Data availability

All of the data that support the findings of this study are available in the main text.
